# Among the Colleges

**Published:** 1902-08

**Authors:** 


### AMONG THE COLLEGES.

THE annual announcement of the Ontario Veterinary College, session 1902–3 has been received. The session will commence this year on October 15th. The only changes noted are the addition to the staff of professors the name of W. J. R. Fowler, V.S., as assistant demonstrator of anatomy, and to the text-books used that of *Materia Medica*, by Winslow.

The announcement of the opening of a third college in Kansas City, Mo., under the name of the University Veterinary College was a great surprise to the profession. Inasmuch as one three-year college and one two-year institution already existed in that city, neither of which had the support or strength it should have in these days of veterinary progress and advancement, one can hardly conjecture how a third institution can hope for such support as will insure its integrity and worth to the true growth of the profession. On the contrary, we fear it is purely a rival institution, and will tend only to injure the cause of veterinary education. So long as State governments will charter such institutions under the corporation laws, true education must suffer until those laws governing educational institutions shall be most severely changed and rigidly enforced.

